# Estimating the impact of vaccination on reducing COVID-19 burden in the United States: December 2020 to March 2022

**DOI:** 10.7189/jogh.12.03062

**Published:** 2022-09-03

**Authors:** Pratha Sah, Thomas N Vilches, Abhishek Pandey, Eric C Schneider, Seyed M Moghadas, Alison P Galvani

**Affiliations:** 1 Center for Infectious Disease Modeling and Analysis (CIDMA), Yale School of Public Health, New Haven, Connecticut, USA; 2 Agent-Based Modelling Laboratory, York University, Toronto, Ontario, Canada; 3 The Commonwealth Fund, New York City, New York, USA

Since the start of COVID-19 vaccination in the United States (US), over 560 million doses of authorized vaccines were administered, and 69.7% of the eligible population were fully vaccinated as of March 31, 2022 [1]. Much attention has focused on the public health toll of the pandemic. The positive impact of the rapid development and deployment of highly efficacious vaccines, ie, the reduction in deaths, hospitalizations, and health care costs, remains unclear. We estimated the reduction in COVID-19 cases, hospitalizations and mortality, as well as averted health care costs achieved by the vaccination program from December 12, 2020 to March 31, 2022.

We expanded an age-stratified, agent-based model of COVID-19 [2] to include waning of vaccine-elicited and naturally-acquired immunity, as well as booster vaccination (Figure S1 in the **Online Supplementary Document**). The epidemiological characteristics of the Iota (B.1.526), Alpha (B.1.1.7), Delta (B.1.617.2), and Omicron (B.1.1.529) variants were included, in addition to the original Wuhan-I SARS-CoV-2 strain (**Online Supplementary Document**). The model was parameterized with the US demographics [3] and age-specific risks of severe health outcomes due to the different variants. We incorporated vaccination based on the reported daily vaccine doses administered to the different age groups [1]. Vaccine efficacies against infection, symptomatic infection, and severe disease for different vaccine types — with regard to each variant and by time since vaccination — were drawn from published estimates (Tables S2 and S3 in the **Online Supplementary Document**). The model was calibrated and fitted to the reported national incidence of COVID-19 per 100 000 capita between October 1, 2020, and March 31, 2022 (**Figure 1**, panel A), and validated with the trends of hospitalizations and deaths during the same period. We also calculated direct health care costs associated with COVID-19 illness with and without vaccination using our projections of COVID-19 symptomatic infections and hospitalizations. Direct costs of health outcomes were stratified into outpatient visits for symptomatic infection, emergency medical services calls, and emergency department visits and hospitalizations for severe illness (Table S4 in the **Online Supplementary Document**) [4-6]. Indirect costs associated with vaccination and disease outcomes (eg, isolation for symptomatic cases) were accounted for by the loss of workdays (Tables S5 and S6 in the **Online Supplementary Document**). Costs related to workdays lost were estimated by considering the percentage of vaccinated adults who are employed and per capita gross domestic product of US$69 288 in the US in 2021. To account for uncertainty in costs, we performed a sensitivity analysis using the Latin Hypercube Sampling technique [7]. Additionally, we took into account the age of individuals at death and the life expectancy at a given age [8] to calculate the years of life lost (YLL) averted by vaccination. To determine the impact of the vaccination program in the US, we simulated the counterfactual scenario of no vaccination and compared the outcome measures generated in the simulated trajectories against the observed measures.

**Figure 1:**
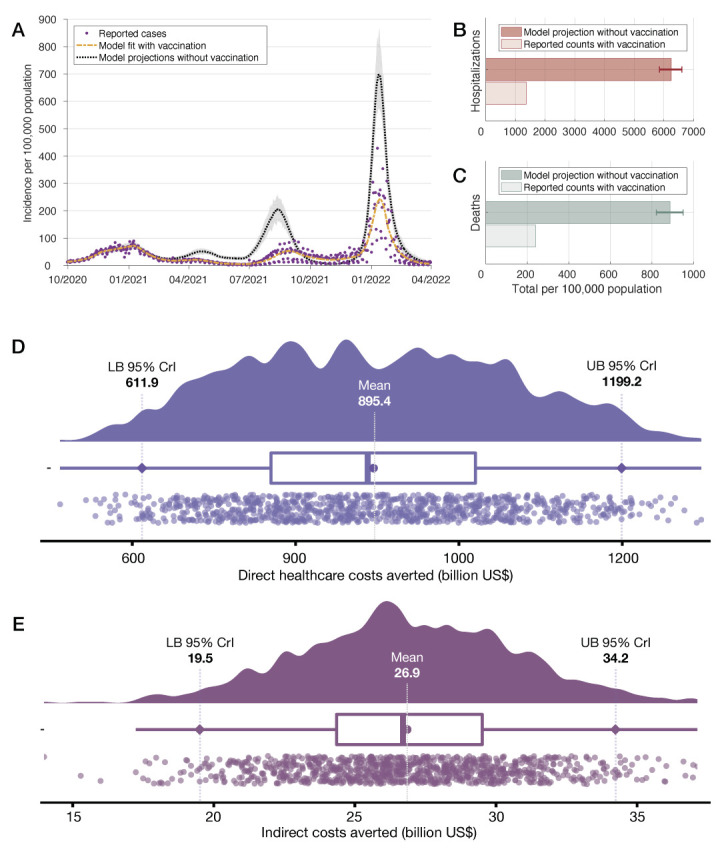
Model fit to the daily national incidence of COVID-19 per 100 000 capita (**Panel A**). Model projections and 95% credible intervals for total hospitalizations (**Panel B**) and deaths (**Panel C**) are compared with the actual toll per 100 000 capita between December 12, 2020, and March 31, 2022. Direct health care costs (**Panel D**) and indirect costs (**Panel E**) averted by vaccination during the same period.

We estimated that COVID-19 vaccination in the US has saved 681 (95% credible Interval (CrI) = 617-742) lives and prevented 5107 (95% CrI = 4709-5481) hospitalizations per 100 000 capita (**Figure 1**, panels B and C). The number of cases averted per 100 000 capita during the same period was projected to be 21 332 (95% CrI = 19 068-23 609). Extrapolating these estimates (**Online Supplementary Document**), we found that COVID-19 vaccination cumulatively averted 2 265 222 (95% CrI = 2 051 041-2 467 683) deaths and prevented 17 003 960 (95% CrI = 15 680 556-18 250 413) hospitalizations. The cumulative YLL averted by vaccination was estimated to be 4998 (95% CrI = 4530-5440) per 100 000 capita and 16 641 801 (95% CrI = 15 083 113-18 112 780) for the country. Vaccination in the US saved US$895.4 (95% CrI = US$611.9-1199.2) billion in direct health care costs and $26.9 (95% CrI = US$19.5-34.2) billion in indirect cost through the end of March 2022 (**Figure 1**, panels D and E).

As of March 2022, COVID-19 has caused over 4.7 million reported hospitalizations with nearly one million documented deaths in the US. Yet, over 60% of deaths caused by the pandemic occurred after the start of the vaccination programme. A significant portion of this burden is attributed to unvaccinated individuals. Our results show that, by March 31, 2022, more lives have been saved by the vaccination program in the US than lost since the beginning of the pandemic. Furthermore, an estimated net saving of nearly $900 billion was achieved in direct costs of the health care system in addition to savings of $27 billion in indirect costs. While our cost estimates do not account for federal or state level investment in vaccination campaigns, we expect the savings would still outweigh these investments. The swift vaccine rollout has been facilitated by the mobilisation of the Federal Emergency Management Agency (FEMA) and national guards, the American Rescue Plan provision of over $200 million to support community-based vaccination efforts, and vaccination incentives and mandates. There has been widespread contention over a recent bill introduced to fund COVID-19 response programs, the COVID Supplemental Appropriation Act [9], which allocates less than $4.25 billion toward the vaccination campaign. The proposed budget is two orders of magnitude less than what we estimate the vaccination campaign has already saved in direct health care costs. In the face of the continued emergence of highly transmissible COVID-19 variants, waning immunity and breakthrough infections, additional funding is needed to bolster vaccination and booster programs that are the cornerstone of pandemic control efforts, to quell future surges in hospitalizations and deaths, leading to even greater health care savings.

**Figure:**
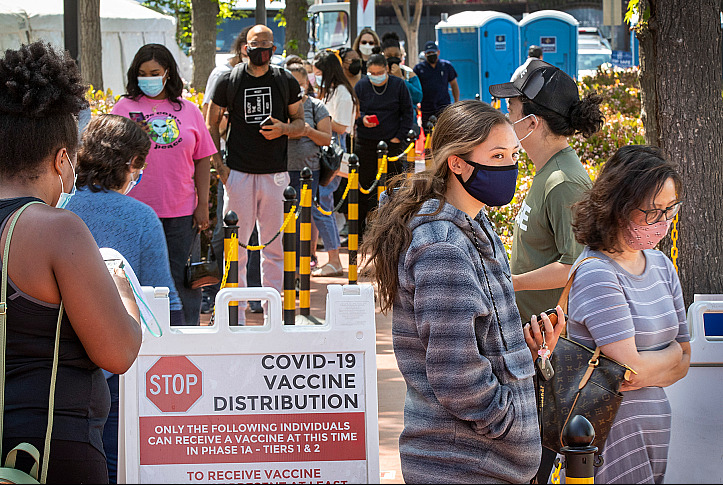
Photo: Courtesy of Commonwealth Fund.

Although heterogeneities and geographic variabilities in COVID-19 outbreaks, interventions, and outcomes were not explicitly included in this modelling study, our results indicate that vaccination in the US has substantially reduced the burden of disease despite the emergence of highly transmissible variants. While the current level of population immunity may prevent the arrival of an Omicron-like wave in the near term, waning immunity and the evolution of SARS-CoV-2 variants with potential for humoral immune evasion necessitate continual investment in vaccination and booster programs.

## Additional material


Online Supplementary Document
