# Supplementary material for: Estimating the impact of vaccination on reducing COVID-19 burden in the United States: December 2020 to March 2022
Source: J Glob Health. 2022 Sep 3;12:03062. doi: 10.7189/jogh.12.03062 (PMC9441009; doi:10.7189/jogh.12.03062)
Supplement: Online Supplementary Document [file jogh-12-03062-s001.pdf]

Appendix S1

Estimating the Impact of Vaccination on Reducing COVID-19 Burden in the United States: December 2020 to March 2022

Pratha Sah[1], PhD, Thomas N. Vilches[1,2], PhD, Abhishek Pandey[1], PhD, Eric C. Schneider[3], MD, Seyed M. Moghadas[2], PhD, Alison P. Galvani[1], PhD

[1] Center for Infectious Disease Modeling and Analysis (CIDMA), Yale School of Public Health, New Haven, Connecticut, USA
[2] Agent-Based Modelling Laboratory, York University, Toronto, Ontario, Canada
[3] The Commonwealth Fund, 1 East 75th Street, New York, NY 10021 USA

This Online Supplementary Document provides details of the model structure and parameterizations for the results reported.

Model structure

We employed our previous agent-based model of COVID-19 transmission[1] and expanded its dynamic structure to account for waning of naturally-acquired or vaccine-elicited immunity, as well as booster vaccination. The model implemented natural history of COVID-19 with epidemiological classes of individuals as susceptible; latently infected (not yet infectious); asymptomatic (and infectious); pre-symptomatic (and infectious); symptomatic (and infectious) with either mild or severe illness; recovered; and dead.

The population was stratified into seven age groups of 0 to 4, 5 to 11, 12-17, 18 to 49, 50 to 64, 65 to 79, and 80+ years based on the US demographics,[2] and incorporated age-specific risk of hospitalizations and deaths, contact patterns. Daily contacts between individuals were sampled from age-specific negative-binomial distributions with parameters that accounted for the effect of interventions such as isolation of symptomatic cases (Table S1).

Table S1. Mixing patterns and the daily number of contacts derived from empirical observations.[3,4] Daily numbers of contacts were sampled from negative binomial distributions for different scenarios.

Age group	Proportion of contacts between age groups					No. of daily contacts Mean (SD)	No. of daily contacts for isolated individuals Mean (SD)
	0-4	5-19	20-49	50-65	65+		
0-4	0.24	0.18	0.42	0.11	0.05	10.21 (7.65)	2.86 (2.14)
5-19	0.02	0.59	0.28	0.08	0.03	16.79 (11.72)	4.70 (3.28)
20-49	0.03	0.16	0.62	0.14	0.05	13.79 (10.50)	3.86 (2.95)
50-65	0.02	0.11	0.48	0.27	0.12	11.26 (9.59)	3.15 (2.66)

65+	0.02	0.11	0.40	0.22	0.25	8.00 (6.96)	2.24 (1.95)

SARS-CoV-2 variants

We considered the spread of four variants, including Iota (B.1.526), Alpha (B.1.1.7), Delta (B.1.617.2), and Omicron (B.1.1.529), in addition to the original Wuhan-I SARS-CoV-2 strain. All variants were introduced in the model at a date corresponding to twice the average duration of their incubation period before the date of identification reported in the GISAID database.[5] Specifically, the Iota variant was introduced on October 25, 2020, with an estimated 35% higher transmissibility compared with the original Wuhan-I strain.[6] We then introduced the Alpha variant on November 29, 2020, with a 50% higher transmissibility compared to the original strain.[7,8] The Delta variant was inserted on February 10, 2021, with an elevated transmissibility of 30% compared with the Alpha variant.[9,10] Finally, we introduced the Omicron variant on November 15, 2021, with a 35% higher transmissibility of Omicron compared to Delta.[11]

Distribution of disease stages and infectiousness

The incubation period for each infected individual was sampled from a log-normal distribution with a mean of 5.2 days.[12] A proportion of infected individuals progressed to a pre-symptomatic stage[13,14] with an infectious period which was sampled from a Gamma distribution with a mean of 2.3 days.[13,15] The symptomatic disease following the pre-symptomatic stage had an average infectious period of 3.2 days, which was also sampled from a Gamma distribution.[13,15] The infectious period of individuals who remained asymptomatic was sampled from a Gamma distribution with a mean of 5 days.[13,16]

Infectiousness was assumed to be highest during the pre-symptomatic stage. The transmissibilities during asymptomatic, mild symptomatic, and severe symptomatic stages were 26%, 44%, and 89%, respectively, relative to the pre-symptomatic stage.[15,17,18]

Disease outcomes

We assumed that asymptomatic and mild symptomatic individuals recover without hospitalization. Self-isolation was implemented to start within 24 hours of symptom onset for all symptomatic individuals, reducing their number of daily contacts by an average of 74% (Table S1). Severely ill individuals due to primary infection were hospitalized within 2-5 days of symptom onset, [19,20] and therefore effectively excluded from the chain of disease transmission. The model was parameterized with rates of intensive care unit (ICU) and non-ICU admissions.[21–23] The risk of hospitalization with the Delta variant was assumed to be 2.26 times higher than that due to infection with Alpha.[23] We considered a 75.2% (95% confidence interval: 72.0% – 77.0%) risk reduction of hospitalization for severe disease due to infection by Omicron compared to Delta.[24,25] The risk of ICU admissions was reduced by 38.1% in severe patients of Omicron compared to those infected with Delta.[26]

Vaccination and immune dynamics

The number of vaccine doses per day and distribution of the first and second doses were parameterized with reported vaccination data in different age groups.[27] The booster eligibility

was set to a 6-month period elapsed since the last dose of vaccine in fully vaccinated individuals. On January 3, 2022, this timeline was reduced to 5 months.[28]

We performed a literature review to derive the efficacy estimates following each dose of vaccine against infection, symptomatic disease, and severe disease for all variants in the model. Estimates of vaccine effectiveness for different SARS-CoV-2 variants in the model are summarized in Tables S2, S3. We assumed the same degree of reduced protection in naturallyacquired immunity as vaccine-induced immunity (without booster) against Omicron.

To implement the waning immunity after vaccination, we fitted a Gaussian model to estimates of vaccine effectiveness over time,[29–32] and determined the temporal relative effectiveness curves (Figure S2). The relative effectiveness was used as a multiplicative factor in the efficacy of vaccines after the second dose to determine the temporal immunity of individuals against infection and severe disease for each variant. We applied the same relative effectiveness for waning of naturally-acquired immunity.

Table S2. Estimated vaccine efficacies (%) and their 95% confidence intervals from published studies for Pfizer-BioNTech vaccines. Booster dose restored or increased the protection efficacy of two doses.

**Vaccine efficacy (%)**	**Timelines**		**Reference**
Original strain and Iota variant	1 week after the second dose	1 week after the booster dose	33,34
Infection	86.1 (82.4, 89.1)	86.1 (82.4, 89.1)	
Symptomatic disease	93.0 (88.0, 95.0)	93.0 (88.0, 95.0)	
Severe disease	98.0 (90.0, 99.0)	98.0 (90.0, 99.0)	
Gamma variant			34,35
Infection	75 (70.5, 78.9)	75 (70.5, 78.9)	
Symptomatic disease	82.0 (65.0, 91.0)	82.0 (65.0, 91.0)	
Severe disease	96.0 (68.0, 99.0)	96.0 (68.0, 99.0)	
Alpha variant			34,35

Infection	89.5 (85.9, 92.3)	89.5 (85.9, 92.3)	
Symptomatic disease	89.0 (87.0, 90.0)	89.0 (87.0, 90.0)	
Severe disease	96.0 (94.0, 97.0)	96.0 (94.0, 97.0)	
Delta variant			34,36
Infection	85.0 (79.0, 90.0)	85.0 (79.0, 90.0)	
Symptomatic disease	92.0 (90.0, 94.0)	92.0 (90.0, 94.0)	
Severe disease	97.0 (96.0, 98.0)	97.0 (96.0, 98.0)	
Omicron variant			37,38
Infection	33.0 (31.0, 35.0)	76.0 (72.0, 79.0)	
Symptomatic disease	69 (62.0, 75.0)	82.0 (79.0, 84.0)	
Severe disease	81.0 (65.0, 90.0)	90.0 (80.0, 94.0)	

Table S3. Estimated vaccine efficacies (%) and their 95% confidence intervals from published studies for Moderna vaccines. Booster dose restored or increased the protection efficacy of two doses.

Vaccine efficacy (%)	Timelines		Reference
Original strain and Iota variant	1 week after the second dose	1 week after the booster dose	34,39

Infection	96.4<br>(91.2, 98.5)	96.4<br>(91.2, 98.5)	
Symptomatic disease	96.0<br>(85.0, 99.0)	96.0<br>(85.0, 99.0)	
Severe disease	97.0<br>(78.0, 100.0)	97.0<br>(78.0, 100.0)	
Gamma variant			[34,40]
Infection	77.0<br>(63.0, 86.0)	77.0<br>(63.0, 86.0)	
Symptomatic disease	89.0<br>(21.0, 98.0)	89.0<br>(21.0, 98.0)	
Severe disease	95.0<br>(63, 99)	95.0<br>(63, 99)	
Alpha variant			[34,39]
Infection	98.4<br>(96.9, 99.1)	98.4<br>(96.9, 99.1)	
Symptomatic disease	92.0<br>(88.0, 95.0)	92.0<br>(88.0, 95.0)	
Severe disease	95.0<br>(92.0, 97.0)	95.0<br>(92.0, 97.0)	
Delta variant			[34,41]
Infection	86.7<br>(84.3, 88.7)	94.0<br>(92.3, 95.4)	
Symptomatic disease	95.0<br>(91.0, 97.0)	95.0<br>(91.0, 97.0)	
Severe disease	98.0<br>(93.0, 99.0)	98.0<br>(93.0, 99.0)	
Omicron variant			[41,42]

Infection	42.8 (33.8, 50.7)	67.7 (65.5, 69.7)	
Symptomatic disease	69 (62.0, 75.0)	82.0 (79.0, 84.0)	
Severe disease	81.0 (65.0, 90.0)	90.0 (80.0, 94.0)	

Direct healthcare costs

We used estimates of COVID-19 symptomatic infections and hospitalizations from the projections of our agent-based model to calculate direct costs associated with COVID-19 illness and hospitalizations (Table S4). Costs of health outcomes were stratified into outpatient visits for symptomatic infection, hospitalizations with or without intensive care, emergency medical services (EMS) calls, and emergency department (ED) visits. We assumed that, on average, 50% of symptomatic, non-hospitalized patients seek outpatient care. We also assumed that each non-hospitalized patient with severe COVID-19 accounts for one ED visit during the course of illness. We calculated the total number of EMS calls to be 2.5 times the number of hospitalized patients.[43]

Table S4. Direct costs (in 2021 US dollars) associated with health outcomes due to COVID-19 illness.

Outcomes of COVID-19 illness	Estimated costs	Range	Reference
Outpatient treatment per visit	$893	$586 —$1,337	44
Hospitalization without ICU (average per patient)	$25,188	$10,979 —$34,896	44
Hospitalization with ICU (average per patient)	$70,098	$41,046 —$102,524	44
ED care (average per visit)	$2200	$623 —$3,102	45,46
EMS transportation	$1,005	$712 —$1,546	47

Indirect effects and costs of vaccination and health outcomes

Indirect costs of vaccination included workdays lost for visiting vaccination clinics and loss of productivity due to adverse reactions to vaccines (Table S5) Published estimates were used for

the prevalence of adverse effects following vaccination. We also considered workdays lost due to isolation of symptomatic cases or hospitalization of severe cases (Table S6) Costs related to workdays lost were estimated by considering the percentage of vaccinated adults who are employed (i.e., ~66.5%, calculated based on the unemployment rate in 2021 and the coverage of fully vaccinated adults) and per capita gross domestic product of $69,288 for 2021 in the US.[48]

Table S5. Indirect effects of vaccination.

Effect	Estimate	Source
Workdays lost to receive vaccination	0.5	[49]
Percentage of Spikevax (Moderna vaccine) recipients with adverse reactions		[50]
After the first dose	51.7%	
After the second dose	74.8%	
Percentage of Comirnaty (Pfizer-BioNTech vaccine) recipients with adverse reactions		[50]
After the first dose	48.0%	
After the second dose	64.2%	
Percentage of Adenovirus-based (Janssen vaccine) recipients with adverse reactions	76%	[51]
Lost workdays due to adverse reactions		[52]
After the first dose	1.66 days	
After the second dose	1.39 days	

Table S6. Indirect effects of health outcomes.

Effect	Estimate	Reference
Workdays lost for symptomatic, nonhospitalized cases	10 days	[53]
Symptomatic days of prior to hospitalization	3.5 days	[54–56]
Median duration of hospital stay without ICU for non-Omicron patients	6 days	
Median duration of hospital stay with ICU for non-Omicron patients	15 days	

Median duration of hospital stay without ICU for Omicron patients	3 days	
Median duration of hospital stay with ICU for Omicron patients	7 days	

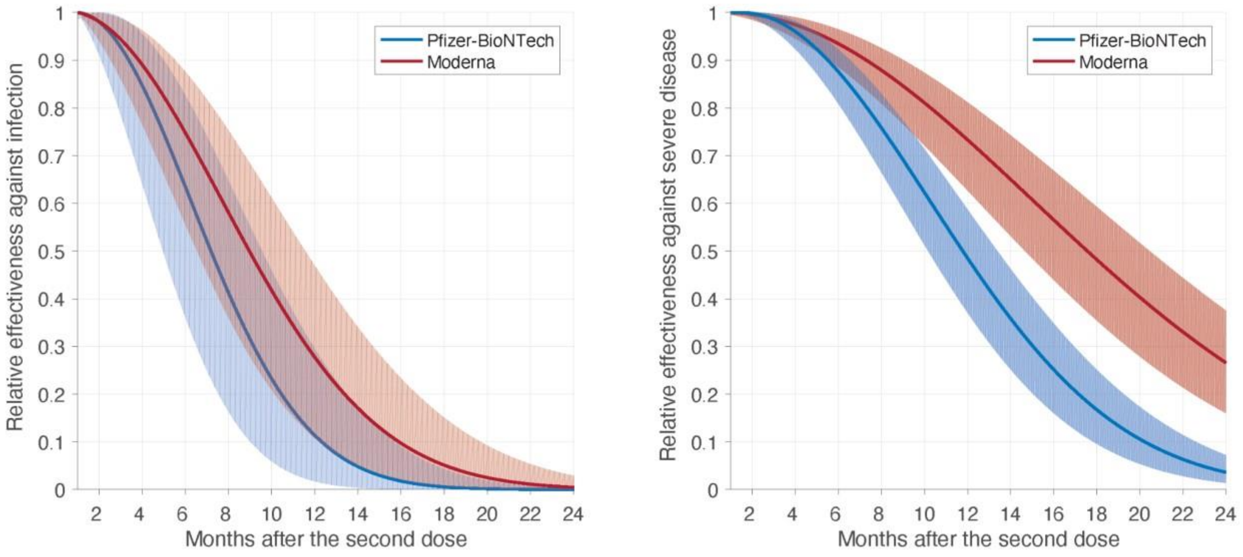

Figure S1. Temporal relative effectiveness of vaccines against infection and severe disease with 95% confidence intervals derived from a Gaussian fit to estimated effectiveness after the second dose of vaccines.[29–31]

Model implementation

With the transmission probability derived from the calibration process, we fitted the model to incidence per 100,000 population from October 1, 2020 to January 31, 2022. For fitting, the agespecific contact rates were adjusted throughout the simulations to minimize the difference between the temporal cumulative incidence predicted by the model and the cumulative reported cases, implicitly accounting for the change and effect of various non-pharmaceutical measures. Simulations were averaged over 500 independent Monte-Carlo realizations, and 95% credible intervals were derived using a bias-corrected and accelerated bootstrap method (with 500 replications), which corrects for bias and skewness in the distribution of bootstrap estimates when scaled from the per capita to the entire US population. We derived model outcomes for symptomatic infections, hospitalizations, and deaths, and calculated the years of life lost (YLL) using the age at death and life expectancy table. The model was implemented in Julia, and simulation codes are available at:
https://github.com/thomasvilches/USomicron/tree/national_estimation

Sensitivity analysis

To account for the range of direct costs (Table S4), we used the Latin Hypercube Sampling technique[57] to generate samples of size 1000 in which each outcome parameter was treated as a random variable and assigned a probability function. The parameters were uniformly distributed and sampled within their respective ranges. We then used these samples to estimate averted costs with outcomes derived from 500 bootstrap replicates of Monte-Carlo simulations.

References

1 Vilches TN, Moghadas SM, Sah P, *et al.* Estimating COVID-19 Infections, Hospitalizations, and Deaths Following the US Vaccination Campaigns During the Pandemic. *JAMA Netw Open* 2022; **5**: e2142725.

2 Explore census data. https://data.census.gov/cedsci/table?q=United%20States&table=DP05&tid=ACSDP1Y2017.DP05&g=0100000US&lastDisplayedRow=29&vintage=2017&layer=state&cid=DP05_0001E (accessed Jan 25, 2022).

3 Mossong J, Hens N, Jit M, *et al.* Social contacts and mixing patterns relevant to the spread of infectious diseases. *PLoS Med* 2008; **5**: e74.

4 Jarvis CI, Van Zandvoort K, Gimma A, *et al.* Quantifying the impact of physical distance measures on the transmission of COVID-19 in the UK. *BMC Med* 2020; **18**: 124.

5 Outbreak.Info. outbreak.info. https://outbreak.info/location-reports?loc=USA&dark=true (accessed March 28, 2022).

6 Annavajhala MK, Mohri H, Wang P, *et al.* Emergence and Expansion of the SARS-CoV-2 Variant B.1.526 Identified in New York. *medRxiv* 2021; published online Aug 4. DOI:10.1101/2021.02.23.21252259.

7 Davies NG, Abbott S, Barnard RC, *et al.* Estimated transmissibility and impact of SARShttp://paperpile.com/b/vVfQRc/uEAbCoV-2 lineage B.1.1.7 in England. *Science* 2021; **372**. DOI:10.1126/science.abg3055.

8 Davies NG, Jarvis CI, CMMID COVID-19 Working Group, *et al.* Increased mortality in community-tested cases of SARS-CoV-2 lineage B.1.1.7. *Nature* 2021; published online March 15. DOI:10.1038/s41586-021-03426-1.

9 Allen H, Vusirikala A, Flannagan J, *et al.* Household transmission of COVID-19 cases associated with SARS-CoV-2 delta variant (B.1.617.2): national case-control study. *Lancet Reg Health Eur* 2022; **12**: 100252.

10 Dhar MS, Marwal R, Vs R, *et al.* Genomic characterization and epidemiology of an emerging SARS-CoV-2 variant in Delhi, India. *Science* 2021; **374**: 995–9.

11 Yang W, Shaman J. SARS-CoV-2 transmission dynamics in South Africa and epidemiological characteristics of the Omicron variant. *medRxiv* 2021; published online Dec 21. DOI:10.1101/2021.12.19.21268073.

12 Li Q, Guan X, Wu P, *et al.* Early Transmission Dynamics in Wuhan, China, of Novel Coronavirus–Infected Pneumonia. *N Engl J Med* 2020; **382**: 1199–207.

13 Li R, Pei S, Chen B, *et al.* Substantial undocumented infection facilitates the rapid dissemination of novel coronavirus (SARS-CoV-2). *Science* 2020; **368**: 489–93.

14 Sah P, Fitzpatrick MC, Zimmer CF, *et al.* Asymptomatic SARS-CoV-2 infection: A systematic review and meta-analysis. *Proc Natl Acad Sci U S A* 2021; **118**. DOI:10.1073/pnas.2109229118.

15 Moghadas SM, Fitzpatrick MC, Sah P, *et al.* The implications of silent transmission for the control of COVID-19 outbreaks. *Proc Natl Acad Sci U S A* 2020; **117**: 17513–5.

16 Gatto M, Bertuzzo E, Mari L, *et al.* Spread and dynamics of the COVID-19 epidemic in Italy: Effects of emergency containment measures. *Proc Natl Acad Sci U S A* 2020; **117**: 10484–91.

17 Ferretti L, Wymant C, Kendall M, *et al.* Quantifying SARS-CoV-2 transmission suggests epidemic control with digital contact tracing. *Science* 2020; **368**. DOI:10.1126/science.abb6936.

18 Sayampanathan AA, Heng CS, Pin PH, Pang J, Leong TY, Lee VJ. Infectivity of asymptomatic versus symptomatic COVID-19. *Lancet* 2021; **397**: 93–4.

19 Shoukat A, Wells CR, Langley JM, Singer BH, Galvani AP, Moghadas SM. Projecting demand for critical care beds during COVID-19 outbreaks in Canada. *CMAJ* 2020; **192**: E489–96.

20 Moghadas SM, Shoukat A, Fitzpatrick MC, *et al.* Projecting hospital utilization during the COVID-19 outbreaks in the United States. *Proc Natl Acad Sci U S A* 2020; **117**: 9122–6.

21 Garg S, Kim L, Whitaker M, *et al.* Hospitalization Rates and Characteristics of Patients Hospitalized with Laboratory-Confirmed Coronavirus Disease 2019 - COVID-NET, 14 States, March 1-30, 2020. *MMWR Morb Mortal Wkly Rep* 2020; **69**: 458–64.

22 Team CC-19 R, CDC COVID-19 Response Team, Chow N, *et al.* Preliminary Estimates of the Prevalence of Selected Underlying Health Conditions Among Patients with Coronavirus Disease 2019 — United States, February 12–March 28, 2020. MMWR. Morbidity and Mortality Weekly Report. 2020; **69**: 382–6.

23 Twohig KA, Nyberg T, Zaidi A, *et al.* Hospital admission and emergency care attendance risk for SARS-CoV-2 delta (B.1.617.2) compared with alpha (B.1.1.7) variants of concern: a cohort study. *Lancet Infect Dis* 2021; published online Aug 27. DOI:10.1016/S1473http://dx.doi.org/10.1016/S1473-3099(21)00475-83099(21)00475-8.

24 Scribner H. Omicron variant leads to less severe symptoms, deaths, new study says. Deseret News. 2021; published online Dec 31. https://www.deseret.com/coronavirus/2021/12/31/22861222/omicron-variant-less-severehttps://www.deseret.com/coronavirus/2021/12/31/22861222/omicron-variant-less-severe-covid-symptoms-deathscovid-symptoms-deaths (accessed Jan 6, 2022).

25 UK Health Security Agency. Technical briefing: Update on hospitalisation and vaccine effectiveness for Omicron VOC-21NOV-01 (B.1.1.529). 2021 https://assets.publishing.service.gov.uk/government/uploads/system/uploads/attachment_data/file/1045619/Technical-Briefing-31-Dec-2021-Omicron_severity_update.pdf.

26 Maslo C, Friedland R, Toubkin M, Laubscher A, Akaloo T, Kama B. Characteristics and Outcomes of Hospitalized Patients in South Africa During the COVID-19 Omicron Wave Compared With Previous Waves. *JAMA* 2021; published online Dec 30. DOI:10.1001/jama.2021.24868.

27 CDC. COVID data tracker. Centers for Disease Control and Prevention. 2020; published online March 28. https://covid.cdc.gov/covid-data-tracker/ (accessed Dec 16, 2021).

28 Office of the Commissioner. Coronavirus (COVID-19) update: FDA takes multiple actions to expand use of Pfizer-BioNTech COVID-19 vaccine. U.S. Food and Drug Administration. 2022; published online Jan 3. https://www.fda.gov/news-events/presshttps://www.fda.gov/news-events/press-announcements/coronavirus-covid-19-update-fda-takes-multiple-actions-expand-use-pfizer-biontech-covid-19-vaccineannouncements/coronavirus-covid-19-update-fda-takes-multiple-actions-expand-use-pfizerhttps://www.fda.gov/news-events/press-announcements/coronavirus-covid-19-update-fda-takes-multiple-actions-expand-use-pfizer-biontech-covid-19-vaccinebiontech-covid-19-vaccine (accessed Jan 6, 2022).

29 Cohn BA, Cirillo PM, Murphy CC, Krigbaum NY, Wallace AW. SARS-CoV-2 vaccine protection and deaths among US veterans during 2021. *Science* 2021; : eabm0620.

30 Tartof SY, Slezak JM, Fischer H, *et al.* Effectiveness of mRNA BNT162b2 COVID-19 vaccine up to 6 months in a large integrated health system in the USA: a retrospective cohort study. *Lancet* 2021; **398**: 1407–16.

31 Chemaitelly H, Yassine HM, Benslimane FM, *et al.* mRNA-1273 COVID-19 vaccine effectiveness against the B.1.1.7 and B.1.351 variants and severe COVID-19 disease in Qatar. Nature Medicine. 2021; **27**: 1614–21.

32 Wright BJ, Tideman S, Diaz GA, French T, Parsons GT, Robicsek A. Comparative vaccine effectiveness against severe COVID-19 over time in US hospital administrative data: a case-control study. *Lancet Respir Med* 2022; published online Feb 25. DOI:10.1016/S2213http://dx.doi.org/10.1016/S2213-2600(22)00042-X2600(22)00042-X.

33 Dagan N, Barda N, Kepten E, *et al.* BNT162b2 mRNA Covid-19 Vaccine in a Nationwide Mass Vaccination Setting. *N Engl J Med* 2021; **384**: 1412–23.

34 Nasreen S, Chung H, He S, *et al.* Effectiveness of COVID-19 vaccines against symptomatic SARS-CoV-2 infection and severe outcomes with variants of concern in Ontario. *Nat Microbiol* 2022; published online Feb 7. DOI:10.1038/s41564-021-01053-0.

35 Abu-Raddad LJ, Chemaitelly H, Butt AA, National Study Group for COVID-19 Vaccination. Effectiveness of the BNT162b2 Covid-19 Vaccine against the B.1.1.7 and B.1.351 Variants. *N Engl J Med* 2021; published online May 5. DOI:10.1056/NEJMc2104974.

36 Pouwels KB, Pritchard E, Matthews PC, *et al.* Effect of Delta variant on viral burden and vaccine effectiveness against new SARS-CoV-2 infections in the UK. *Nat Med* 2021; **27**: 2127–35.

37 Andeweg SP, de Gier B, Eggink D, *et al.* Protection of COVID-19 vaccination and previous infection against Omicron BA.1 and Delta SARS-CoV-2 infections, the Netherlands, 22 November 2021- 19 January 2022. bioRxiv. 2022; published online Feb 7. DOI:10.1101/2022.02.06.22270457.

38 Ferdinands JM. Waning 2-Dose and 3-Dose Effectiveness of mRNA Vaccines Against COVID-19–Associated Emergency Department and Urgent Care Encounters and Hospitalizations Among Adults During Periods of Delta and Omicron Variant Predominance — VISION Network, 10 States, August 2021–January 2022. *MMWR Morb Mortal Wkly Rep* 2022; **71**. DOI:10.15585/mmwr.mm7107e2.

39 Bruxvoort KJ, Sy LS, Qian L, *et al.* Effectiveness of mRNA-1273 against delta, mu, and other emerging variants of SARS-CoV-2: test negative case-control study. *BMJ* 2021; **375**: e068848.

40 Charmet T, Schaeffer L, Grant R, *et al.* Impact of original, B.1.1.7, and B.1.351/P.1 SARShttp://paperpile.com/b/vVfQRc/eeaM4CoV-2 lineages on vaccine effectiveness of two doses of COVID-19 mRNA vaccines:
Results from a nationwide case-control study in France. *Lancet Reg Health Eur* 2021; **8**: 100171.

41 Tseng HF, Ackerson BK, Luo Y, *et al.* Effectiveness of mRNA-1273 against SARS-CoV-2 omicron and delta variants. bioRxiv. 2022; published online Jan 8. DOI:10.1101/2022.01.07.22268919.

42 Thompson MG, Natarajan K, Irving SA, *et al.* Effectiveness of a Third Dose of mRNA Vaccines Against COVID-19-Associated Emergency Department and Urgent Care Encounters and Hospitalizations Among Adults During Periods of Delta and Omicron Variant Predominance - VISION Network, 10 States, August 2021-January 2022. *MMWR Morb Mortal Wkly Rep* 2022; **71**: 139–45.

43 Xie Y, Kulpanowski D, Ong J, Nikolova E, Tran NM. Predicting Covid-19 emergency medical service incidents from daily hospitalisation trends. *Int J Clin Pract* 2021; **75**: e14920.

44 Fair Health. COVID-19 Medical and Hospitalization Costs: National. {Fair Health, Inc}, 2021 https://s3.amazonaws.com/media2.fairhealth.org/infographic/asset/COVID-19%20Medical%20Hospitalization%20Costs%20by%20State%20-%20FINAL_National.pdf.

45 Urgent care vs. ER. https://www.uhc.com/member-resources/where-to-go-for-medicalhttps://www.uhc.com/member-resources/where-to-go-for-medical-care/urgent-care-vs-ercare/urgent-care-vs-er (accessed April 9, 2022).

46 Corso A. Emergency room visit cost without insurance in 2022. Mira. 2021; published online March 2. https://www.talktomira.com/post/how-much-does-an-er-visit-cost (accessed July 28, 2022).

47 Chhabra KR, McGuire K, Sheetz KH, Scott JW, Nuliyalu U, Ryan AM. Most Patients Undergoing Ground And Air Ambulance Transportation Receive Sizable Out-Of-Network Bills. *Health Aff* 2020; **39**: 777–82.

48 Personal income in New York - income per capita 2000-2020. Statista. https://www.statista.com/statistics/205461/per-capita-personal-income-in-new-york/ (accessed March 24, 2022).

49 Bologna JM, Greenberg RI, Skubas SR, Shapiro © Jackson Lewis H. New York employees are entitled to paid time off for COVID-19 vaccinations. SHRM. 2021; published online March 18. https://www.shrm.org/ResourcesAndTools/legal-and-compliance/state-and-localhttps://www.shrm.org/ResourcesAndTools/legal-and-compliance/state-and-local-updates/Pages/New-York-Employees-Are-Entitled-to-Paid-Time-Off-for-COVID-19-Vaccinations.aspxupdates/Pages/New-York-Employees-Are-Entitled-to-Paid-Time-Off-for-COVID-19https://www.shrm.org/ResourcesAndTools/legal-and-compliance/state-and-local-updates/Pages/New-York-Employees-Are-Entitled-to-Paid-Time-Off-for-COVID-19-Vaccinations.aspxVaccinations.aspx (accessed March 20, 2022).

50 Chapin-Bardales J, Gee J, Myers T. Reactogenicity Following Receipt of mRNA-Based COVID-19 Vaccines. *JAMA* 2021; **325**: 2201–2.

51 Shay DK, Gee J, Su JR, *et al.* Safety Monitoring of the Janssen (Johnson & Johnson) COVID-19 Vaccine - United States, March-April 2021. *MMWR Morb Mortal Wkly Rep* 2021; **70**: 680–4.

52 Levi ML, McMillan D, Dhandha V, Allan J, D'ercole F. COVID-19 mRNA vaccination, reactogenicity, work-related absences and the impact on operating room staffing: A crosshttp://paperpile.com/b/vVfQRc/XZmBOsectional study. *Perioper Care Oper Room Manag* 2021; **25**: 100220.

53 COVID-19: What to do when sick. https://www1.nyc.gov/site/doh/covid/covid-19-symptomshttps://www1.nyc.gov/site/doh/covid/covid-19-symptoms-chronic-health-risks.pagechronic-health-risks.page (accessed March 11, 2022).

54 Iuliano AD, Brunkard JM, Boehmer TK, *et al.* Trends in Disease Severity and Health Care Utilization During the Early Omicron Variant Period Compared with Previous SARS-CoV-2 High Transmission Periods - United States, December 2020-January 2022. *MMWR Morb Mortal Wkly Rep* 2022; **71**: 146–52.

55 Christensen PA, Olsen RJ, Long SW, *et al.* Signals of Significantly Increased Vaccine Breakthrough, Decreased Hospitalization Rates, and Less Severe Disease in Patients with Coronavirus Disease 2019 Caused by the Omicron Variant of Severe Acute Respiratory Syndrome Coronavirus 2 in Houston, Texas. *Am J Pathol* 2022; published online Feb 3. DOI:10.1016/j.ajpath.2022.01.007.

56 Nguyen NT, Chinn J, Nahmias J, *et al.* Outcomes and Mortality Among Adults Hospitalized With COVID-19 at US Medical Centers. *JAMA Netw Open* 2021; **4**: e210417.

57 McKay MD, Beckman RJ, Conover WJ. A Comparison of Three Methods for Selecting Values of Input Variables in the Analysis of Output from a Computer Code. *Technometrics* 1979; **21**: 239–45.
